# Recurrent intussusception associated with pneumatosis cystoides coli: A pediatric case report

**DOI:** 10.1002/jpr3.70109

**Published:** 2025-11-14

**Authors:** Sarah Barrett, Tanyaporn Kaenkumchorn, Pooja Thakrar, Rosellen Choi, Rose Lee

**Affiliations:** ^1^ Division of Gastroenterology Medical College of Wisconsin Milwaukee Wisconsin USA; ^2^ Department of Radiology Medical College of Wisconsin Milwaukee Wisconsin USA; ^3^ Division of Hospital Medicine Medical College of Wisconsin Milwaukee Wisconsin USA

**Keywords:** abdominal pain, hyperbaric oxygen therapy, pediatrics

## Abstract

Pneumatosis cystoides coli (PCC) describes gas‐filled cysts within the wall of the gastrointestinal tract and is uncommon in children. We report a 7‐year‐old female with a history of recurrent ileocolic intussusception secondary to PCC. Her initial episodes of intussusception resolved spontaneously or with air enema, but no lead point was identified. On her third presentation to the emergency department with a similar complaint, computed tomography of the abdomen and pelvis revealed extensive colonic mural air‐filled cystic lesions which had been present during prior episodes of intussusception. The patient was treated with metronidazole, a sorbitol‐free diet, and continuous low‐flow oxygen. Despite these treatments, PCC persisted on imaging. Hyperbaric oxygen treatment was initiated, leading to complete resolution of PCC without any further episodes.

## 1 INTRODUCTION

Pneumatosis cystoides coli (PCC) is a rare condition defined by the presence of gas‐filled cysts within the colon wall. Most cases of PCC are provoked; only up to 15% of cases occur spontaneously.^1^ The two primary mechanisms by which intraluminal gas can be introduced into the bowel wall are an increase in hydrogen‐producing bacteria in the intestine and increased intraluminal pressure that forces air into the bowel wall.^1^ Patients with compromised bowel wall integrity, such as those with immunodeficiencies or inflammatory bowel disease, are at an increased risk of developing PCC.^2^ PCC occurs most frequently in adults affected by these conditions or in neonates with necrotizing enterocolitis. It rarely affects healthy children.^3^

PCC is often an asymptomatic finding identified incidentally on imaging or autopsy. However, it can lead to serious complications such as intussusception, bowel obstruction, or bowel perforation.^1^ Early intervention is essential to prevent these potential life‐threatening complications. Management focuses on reducing gas production in the bowel through bowel rest and antibiotics and reabsorbing mural gas through the use of supplemental oxygen and hyperbaric oxygen therapy (HBO).^2^

HBO has been well documented as a treatment for PCC in adults.^4^, ^5^, ^6^, ^7^ However, the use of HBO to treat PCC in children is rarely discussed in the literature. We present a case which demonstrates the use of HBO as an effective and well‐tolerated treatment for PCC in a 7‐year‐old female.

## 2 CASE REPORT

**Figure 1:**
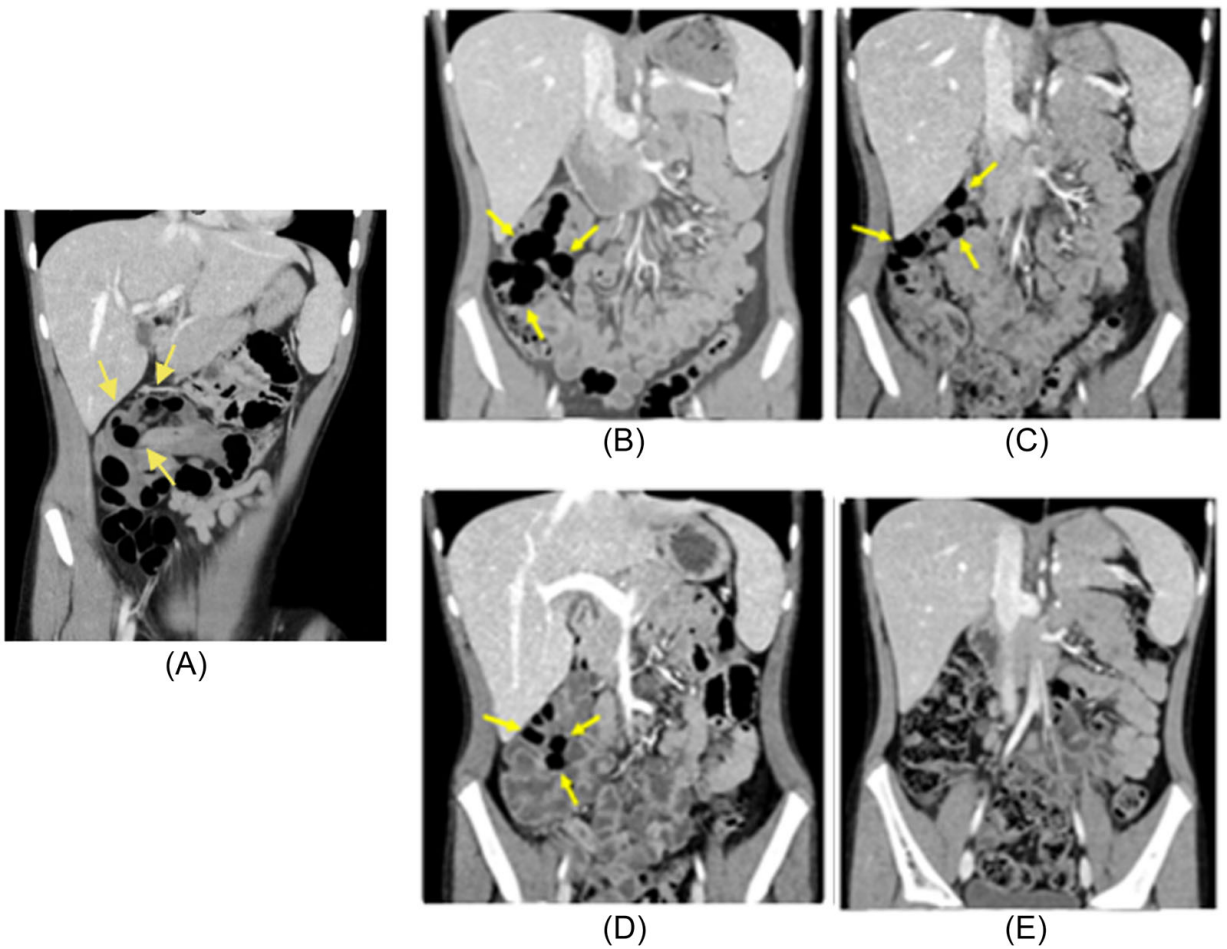
CT scans of the patient's abdomen throughout the course of treatment. Yellow arrows indicate PCC. (A) Initial CT obtained 10 months before admission. (B) Day of admission. (C) Day 10 of treatment with metronidazole and supplemental oxygen. (D) Day 28 of treatment, after 4 days of hyperbaric oxygen therapy. (E) Complete resolution of PCC 6 months after hospital admission. CT, computed tomography; PCC, pneumatosis cystoides coli.

A 7‐year‐old female with a history of recurrent ileocolic intussusception presented with 1 day of bilious emesis. She had two prior episodes of intussusception, both confirmed by ultrasound. The first resolved with air enema, the second spontaneously. No lead point was identified on abdominal computed tomography (CT) performed before any intervention or during exploratory laparotomy. During her third episode, the patient presented with 1 day of bilious emesis and abdominal pain. Ultrasound confirmed ileocolic intussusception, which was reduced with a water‐soluble contrast enema, and she was discharged. She returned the next day with recurrent symptoms and diarrhea. CT revealed cystic pneumatosis coli involving the ascending colon, hepatic flexure, and proximal to mid transverse colon, with the largest cyst measuring 3.6 cm (Figure 1). Retrospective review of her initial CT showed these cysts were present but misinterpreted as intraluminal gas.

**Figure 2:**
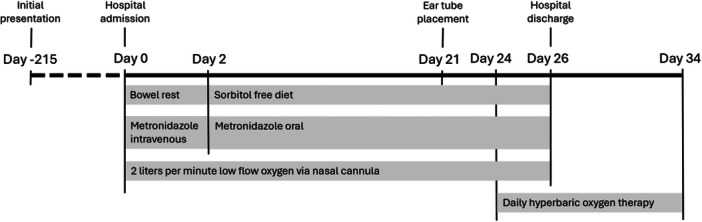
Timeline of therapy from initial two presentations of intussusception (resolved with air enema and spontaneously, respectively) to termination of hyperbaric oxygen therapy.

The patient received 2 days of bowel rest, followed by 24 days on a sorbitol‐free diet, 26 days of metronidazole (initially IV, then oral), and low‐flow oxygen at 2 L/min by nasal cannula (Figure 2). Despite this, repeat CT showed persistent PCC. HBO was then initiated, consisting of 10 daily 90‐min sessions at 45 FSW (feet of sea water). Bilateral tympanostomy tubes were placed beforehand to prevent otalgia. She tolerated HBO without side effects.

She remained asymptomatic without intussusception recurrence throughout her admission. Serial CT scans showed significant reduction in the size of the cysts following initiation of HBO. A CT obtained 6 months after completion of treatment revealed resolution of PCC.

Alternate causes of PCC, including celiac disease and lymphoma, were ruled out through lab workup (tissue transglutaminase, complete blood count, uric acid, lactate dehydrogenase, and peripheral blood smear). Imaging showed no lymphadenopathy or pulmonary disease. The initial CT showing PCC was done before air enema or surgery, excluding colonic manipulation as the cause.

In the 8 months following discharge, the patient presented to the emergency department with three isolated episodes of abdominal pain. Abdominal CT scans, abdominal ultrasounds, and esophagogastroduodenoscopy and colonoscopy have shown no evidence of PCC and were otherwise unremarkable. Her abdominal pain has been determined to be due to constipation and disorder of gut–brain interaction and has improved with constipation treatment.

## 3 DISCUSSION

This case demonstrates a safe, effective treatment for PCC in a young child. The theory behind the treatment was twofold. First, antibiotics, bowel rest, and a sorbitol‐free diet help reduce intestinal gas by eliminating gas‐producing bacteria and minimizing intestinal gas production during digestion.^8^ Second, HBO promotes gas reabsorption within the cysts by increasing arterial oxygen tension. This drives oxygen diffusion from the bloodstream into the cysts—areas typically low in oxygen and high in hydrogen—displacing hydrogen into the circulation. The delivered oxygen is then absorbed by surrounding cells and used in cellular metabolism, aiding in cyst resolution.^8^

The size of our patient's colonic mural cysts decreased slightly with conservative therapies including low‐flow oxygen, dietary modifications, and antibiotics. However, rapid, complete resolution of the PCC was seen after initiation of HBO. Our patient tolerated HBO without side effects.

HBO is generally well tolerated in children and has been used to treat conditions like carbon monoxide poisoning, decompression sickness, and intracranial abscesses.^9^ Ear discomfort from pressure changes is a common side effect.^10^ Although the patient was low risk, prophylactic tympanostomy tubes were placed to prevent otalgia and ensure treatment compliance.

With no established treatment protocols, duration was guided by imaging and shared decision‐making with the family. The patient remained hospitalized for symptom monitoring due to risk of recurrent intussusception and bowel ischemia. She was discharged once imaging showed cyst reduction, and both metronidazole and the sorbitol‐free diet were discontinued as their risks outweighed benefits.

Although the patient responded well to HBO, treatment was delayed due to the late diagnosis of PCC, which was only identified on CT. As ultrasound is the standard for diagnosing intussusception, PCC as a lead point may be frequently missed. This case highlights the need for greater awareness of PCC in pediatric intussusception and the importance of considering additional imaging in recurrent cases of intussusception.

## 4 CONCLUSION

Although children are generally less susceptible to PCC, people of any age with underlying conditions such as cerebral palsy or immunosuppression are at increased risk for recurrent PCC.^3^ The rare but serious complications of PCC, including bowel obstruction, perforation, ischemia, volvulus, and intussusception, necessitate a reliable treatment for PCC in patients of all ages. Our patient's robust and sustained response to HBO can serve as a roadmap for treatment of children with or at risk for recurrent PCC.

## CONFLICT OF INTEREST STATEMENT

The authors declare no conflicts of interest.

## ETHICS STATEMENT

Informed consent for publication of this case report was obtained from the patient's parents.
